# Practitioners' Bookshelf

**Published:** 1892-06-18

**Authors:** 


### PRACTITIONERS' BOOKSHELF.

### THE NINE CIRCLES.*

This little book consists of verbatim extracts from reports of vivisectors, English and Continental, and is designed for the purpose of showing what vivisection is, and to impress the reader with the amount of suffering which many vivisectional experiments undoubtedly entail. Its title, "The Nine Circles of the Hell of the Innocent, Described from the Reports of the Presiding Spirits," sufficiently indicates the spirit in which the work was planned. But, without entering into any arguments for or against vivisection, we feel that works of this character are little short of a libel on many of the authors referred to in the text, and calculated to mislead the public. We heartily wish that the restrictions imposed on the practice in England were enforced abroad, but we cannot forget that in the opinion of the majority of those best able to judge, our physiological and pathological knowledge has been materially advanced as a result of vivisection, and that we are thereby enabled to render more efficient help in relieving the sufferings of humanity and in saving life. We protest, therefore against writings which place before the reader only the deplorable details without full reference to the good object with which most of the experiments are undertaken. We should infinitely regret to find that such details as are here related did not produce a feeling of revulsion in the mind of every man and woman. But the details of many operations of human surgery would be equally revolting to the general public. The end justifies the means adopted in surgery, but whether vivisection is justified by the results sought or obtained will never be determined by such books as the one before us; nevertheless, we reverence the noble sentiments that have prompted Miss Cobbe and her fellow-workers to attack the practice so vehemently.

### LUNACY LAW.†

The Lunacy Acts of 1845 and 1853 could hardly be described as models of perspicuous arrangement, or of instances of that brevity which we are told is the soul of wit; but the Act of 1889 and the Consolidation Act of 1890 are by no means improvements on the old Acts repealed by them. Indeed, the new Acts are in many respects distinctly retrograde and so confused that an Amendment Bill had to be introduced and passed in 1891, the latter being like a pinch of solder on a vessel having as many holes as a colander.

However, we must put up with the Act for a while at least, and in the meantime Mr. Chamier has done his best to digest the various sections and clauses and present them to us in as clear a form as possible, considering the material at his disposal. It would be difficult to conceive a more useful little book on the subject—useful alike to the lawyer who has business relations with the insane; to the magistrate specially appointed under the Act, and to the medical man who has charge of the patient. It is, in one word, a complete *précis* of the law as it now stands, and is well calculated to give a correct idea of the Act to anyone who will read carefully its clearly and cleverly written pages.

Mr. Chamier wisely abstains from expressing either approval or disapproval of the Act; but he gives us an appendix of eighteen closely-printed pages containing the views of the Medical Superintendents of fifteen public asylums. These views are, without exception, condemnatory of the Act, in many cases strongly condemnatory. Dr. Smith, of the Durham County Asylum, has given one of the shortest and most trenchant criticisms we have yet met. He calls it "an Act to prevent the early treatment of the insane, and to hamper all having care of them."

We predict a career of usefulness and success for this little volume.

* "The Nine Circles of the Hell of the Innocent," by G. M. Rhodes, with Preface by Frances Power Cobbe. pp. 162. (Sonnenschein and Co. London.)

† By Daniel Chamier, Barrister-at-Law. pp. 81. Price 1s. 6d. (Effingham Wilson and Co.)

